# Carvedilol ameliorates dexamethasone-induced myocardial injury in rats independent of its action on the α1-adrenergic receptor

**DOI:** 10.1007/s00210-022-02285-5

**Published:** 2022-09-10

**Authors:** Rasha M. S. M. Mohamed, Enssaf Ahmad Ahmad, Bothina H. F. Omran, Amr T. Sakr, Islam A. A. E.-H. Ibrahim, Mona F. Mahmoud, Mostafa E. El-Naggar

**Affiliations:** 1 Department of Clinical Pharmacology, Faculty of Medicine, Zagazig University, Zagazig, 44519 Egypt; 2 Department of Human Anatomy and Embryology, Faculty of Medicine, Zagazig University, Zagazig, 44519 Egypt; 3 Department of Forensic Medicine and Clinical Toxicology, Faculty of Medicine, Zagazig University, Zagazig, 44519 Egypt; 4 Department of Biochemistry, Faculty of Pharmacy, University of Sadat City, Menoufia, 32897 Egypt; 5 Department of Pharmacology and Toxicology, Faculty of Pharmacy, Zagazig University, Zagazig, 44519 Egypt; 6 Department of Pharmacology and Toxicology, Faculty of Pharmacy, University of Sadat City, Menoufia, 32897 Egypt

**Keywords:** Dexamethasone, Carvedilol, Phenylephrine, Propranolol, Cardiotoxicity

## Abstract

The current study aimed to investigate the cardiotoxic effect of dexamethasone-high-dose in rats, the therapeutic effect of carvedilol and the role of α1-adrenergic receptor (α1AR). The experiment involved 6 groups: control, dexamethasone (10 mg/kg), carvedilol (10 mg/kg), phenylephrine (1 mg/kg), phenylephrine plus carvedilol and propranolol (30 mg/kg). Drugs and vehicles were given for 7 days. Dexamethasone was given with the drugs in the last 4 groups. On the 8^th^-day and after overnight fasting, serum and cardiac samples were collected. Serum levels of cardiac troponin I and creatine kinase–myoglobin as well as cardiac levels of diacylglycerol, malondialdehyde, kinase activity of Akt, transforming growth factor-β, Smad3 and alpha smooth muscle actin were measured. Cardiac samples were also used for histopathological examination using hematoxylin–eosin and Sirius red stains, in addition to immunohistochemical examination using β-arrestin2 antibody. Dexamethasone induced cardiac injury via increasing oxidative stress, apoptosis and profibrotic signals. Carvedilol significantly reduced the dexamethasone-induced cardiotoxicity. Using phenylephrine, a competitive α1-agonist, with carvedilol potentiated the cardioprotective actions of carvedilol. Propranolol, a β-blocker without activity on α1ARs, showed higher cardiac protection than carvedilol. Dexamethasone-high-dose upregulates cardiac oxidative stress, apoptotic and profibrotic signals and induces cardiac injury. Blocking the α1-adrenergic receptor by carvedilol attenuates its cardioprotective effects against dexamethasone-induced cardiotoxicity.

## Introduction

The healthcare data through the period (1996-2010) reported 235,283 cases of both drug-related acute myocardial infarction and non-related ones, with an incidence rate of 153.7 per 100,000 person-year. Several steroidal anti-inflammatory drugs were classified as prime suspects of drug-induced acute myocardial infarction including dexamethasone (Coloma et al. 2013). Dexamethasone is a widely used anti-inflammatory and immunosuppressant medication. In high doses, dexamethasone can induce several adverse effects such as hyperglycemia and cardiac complications (Poetker and Reh 2010). Dexamethasone-induced cardiotoxicity has been reported in preterm infants with respiratory problems and patients with multiple myeloma and multiple sclerosis (Stark et al. 2001; Vasheghani-Farahani et al. 2011; Plummer et al. 2019). The probable mechanisms of dexamethasone-induced cardiotoxicity include atherogenic effects, sensitization of adrenergic receptors and induction of dyslipidemia (Ferrau and Korbonits 2015).

Carvedilol is a third-generation β-blocker with vasodilatory effects mediated by blocking the α1-adrenergic receptors (Frishman 1998). In addition, carvedilol has antioxidant and anti-inflammatory actions and can uniquely activate β-arrestin signaling (Wisler et al. 2007). Carvedilol is clinically used in the treatment of hypertension, angina pectoris and congestive heart failure (Packer et al. 2001; Aronow 2010). The cardioprotective actions of carvedilol have been attributed, at least in part, to the blocking of α1-adrenergic receptors (Poole-Wilson et al. 2003)

The α1-adrenergic receptors are Gαq-protein-coupled receptors which upon stimulation mediate the activation of phospholipase-C (PLC) leading to breakdown of phosphatidyl-inositol 4,5 bisphosphate into diacylglycerol (DAG) and inositol triphosphate (IP3) (Arthur et al. 2001). DAG can activate different down-stream signals involved in the production of reactive oxygen species (ROS), inflammatory, apoptotic and fibrotic signals (Volpe et al. 2018). On the other hand, IP3 increases the intracellular release of calcium from the endoplasmic reticulum which may further contribute to increased apoptosis (Ruiz et al. 2009).

The α1-adrenergic receptors are expressed in different tissues including the vascular smooth muscles and the cardiac muscles. In the blood vessels, activation of α1-adrenergic receptors causes vasoconstriction and hypertension, leading to increased workload on the heart which, in the long-term, can cause cardiac damage (Piascik and Perez 2001). On the contrary and paradoxically, activation of the cardiac α1-adrenergic receptors has been found to reduce cardiomyocyte apoptosis and mediate cardiac protection (Guo et al. 2006). The underlying mechanisms are not clear. Phenylephrine, a selective α1-agonist, has been found to protect the heart against ischemia-reperfusion injury (Jensen et al. 2011). While selective α1-blockers have been found to worsen heart failure even in hypertensive patients (Piller et al. 2002). Therefore, the activation of α1-adrenergic receptors can directly protect the heart and indirectly harm it.

Considering earlier findings, the current study aimed to investigate the cardiotoxic effects of dexamethasone-high-dose in rats. Furthermore, we investigated the possible therapeutic effect of carvedilol against dexamethasone-induced cardiotoxicity and the roles of α1-adrenergic receptor in mediating these effects by using different pharmacological interventions such as phenylephrine (a competitive α1-agonist) and propranolol (a non-selective β-blocker).

## Materials and methods

### Animals

Adult male Wistar albino rats (180 ± 20 g, 8 weeks old) were bought from the Faculty of Veterinary Medicine of our university and housed in plastic cages with wood shave bedding in the animal care unit of our institution. The animals were kept under controlled temperature (23 ± 2 °C), humidity (60% ± 10%) and a 12-h/12-h light/dark cycle. Rats were acclimatized for at least two weeks prior to experiments with ad libitum access to standard pellet chow and tap water.

### Drugs

Dexamethasone and propranolol were obtained from EPICO Co. (10^th^ of Ramadan, Egypt). Carvedilol was obtained from Multi-Apex Pharmaceutical Co. (Cairo, Egypt). Phenylephrine and dimethyl sulfoxide (DMSO) were obtained from Sigma-Aldrich (St. Louis, MO, USA). All chemicals were of the analytical grade.

### Experimental design

**Fig. 1:**
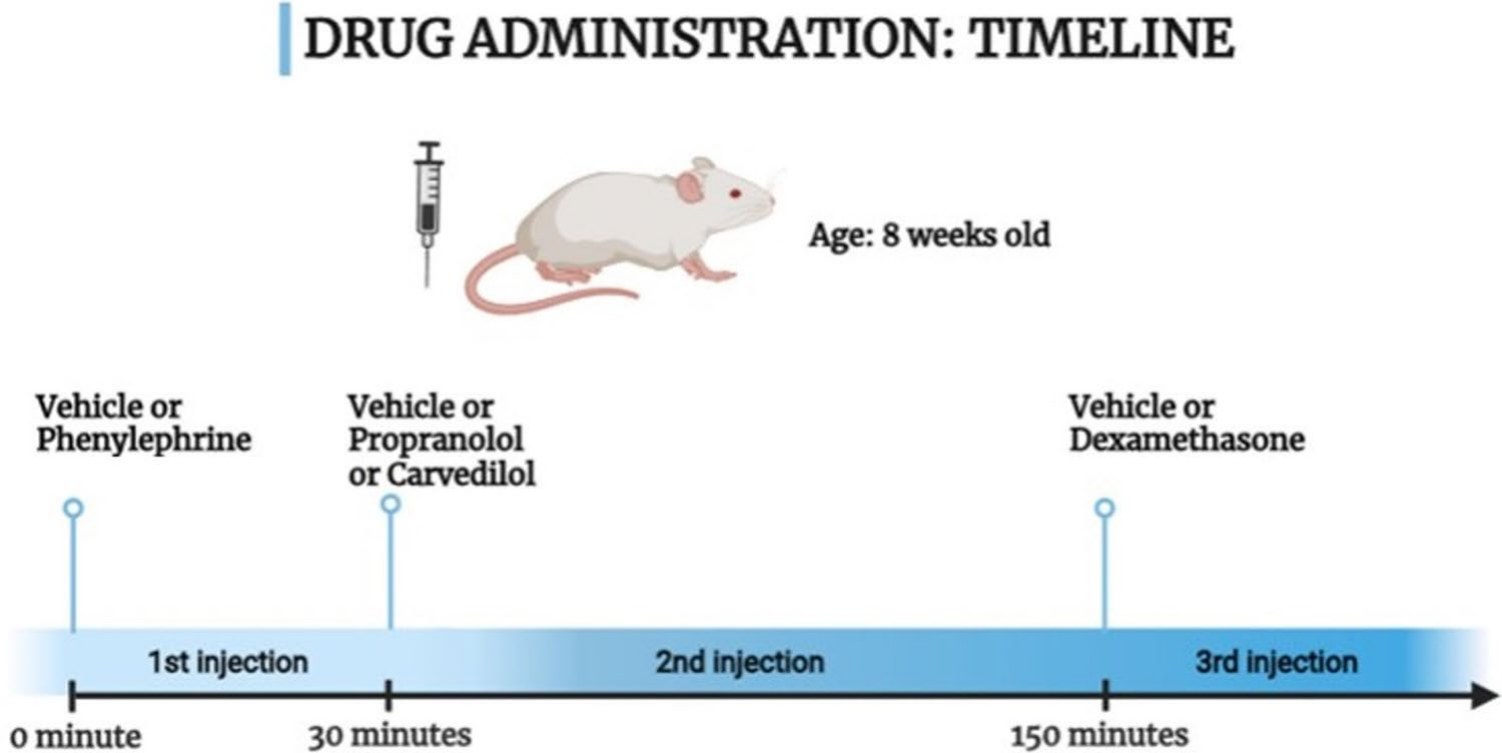
Timeline of drugs and vehicle administration

After the acclimatization period, the experiment was conducted for 7 days. Rats were randomly divided into six experimental groups (9 each). In group 1, rats were given the vehicle (DMSO: Tween 80: Water in a volume ratio of 1:1:8 (El-Fayoumi et al. 2020). In groups 2 to 6, rats were given dexamethasone **[**10 mg/kg/day, subcutaneous (S.C.), (Mahendran and Devi 2001)**]**. In group 3, rats received carvedilol **[**10 mg/kg/day, intraperitoneal (I.P.), (Ibrahim et al. 2020)**]**. In group 4, rats received phenylephrine **[**1 mg/kg/day, I.P., (Hu 2007)**]**. In group 5, rats received Phenylephrine then carvedilol 30 minutes later. In group 6, rats received propranolol **[**30 mg/kg/day, I.P., (Wang et al. 2016)**]**. In all groups, the injection volume was 500 μL per 200g body weight. Drugs and vehicles were given as shown in the diagram in Fig. 1.

### Measurement of heart weights and tibial lengths

At the end of drug treatment, rats were fasted overnight. Tibial lengths were measured. After euthanasia, hearts were dissected and weighted.

### Blood and tissue sampling

At the end of drug treatment, rats were euthanized by decapitation and blood was collected from the site of decapitation. Serum samples were obtained by centrifugation (4000 rpm, 4 °C, 15 min) and stored at −80 °C for subsequent analyses. Heart samples were also collected. Heart samples were divided into two parts; one part was fixed and stored in formalin for histopathological and immunohistochemical examination while the other was snap frozen in liquid nitrogen and stored at −80 °C for later biochemical analyses.

### Determination of serum cardiac troponin I (cTnI) and creatine kinase–myoglobin (CK–MB) concentrations

Serum cTnI and CK–MB were measured by enzyme-linked immunosorbent assays (ELISAs) using kits supplied by MyBioSource (San Diego, USA, Cat. No. MBS727624) and MyBioSource (San Diego, USA; Cat No. MBS008782), respectively. All procedures were performed according to the manufacturer instructions.

### Measurement of biochemical changes in the ventricular cardiac tissue

Levels of diacylglycerol (DAG), Malondialdehyde (MDA), kinase activity of Akt, transforming growth factor β (TGFβ), SMAD3 and alpha smooth muscle actin (α-SMA) were measured in the ventricular cardiac tissue using ELISA kits supplied by MyBioSource (San Diego, USA, Cat. No. MBS750727), LifeSpan BioSciences (Seattle WA, USA, Cat. No. LS-F28018), CD Creative Diagnostics (Shirley NY, USA, Cat. No. DEIABL547), MyBioSource (San Diego, USA, Cat. No. MBS011634), LifeSpan BioSciences (Seattle WA, USA, Cat. No. LS-F21581) and MyBioSource (San Diego, USA, Cat. No. MBS266620), respectively. All procedures were performed according to the manufacturer instructions.

### Histopathological examination

Heart tissue was excised, fixed in 10% formalin, dehydrated in gradient ethanol, cleared in xylene, embedded in paraffin blocks, sectioned at 5-μm thickness and stained with Hematoxylin and Eosin (H&E) stain or Sirius red stain**.**

### Calculation of fibrotic area using Sirius red stain

The relative collagen fiber staining area (% red area) was measured in ventricular sections from three rats in each group, using ImageJ v.1.51d (NIH & LOCI, Wisconsin University, USA). Briefly, fiber-positive (red-colored) areas were selected, masked by red binary coloring and measured on a standard measurement frame.

### Immunohistochemical examination of cardiac β-arrestin2

An immunohistochemical study was performed using the avidin-biotin-peroxidase technique. Briefly, sections of 5μm were deparaffinized, rehydrated, washed in tap water and suspended in 3% H2O2 for 10 min to inhibit the endogenous peroxidase. Trypsin (2%) was added to the tissue sections at 37°C for 10 min to induce the affinity for immune peroxidase staining technique. The tissue sections were placed in a solution of (10 mmol/l sodium citrate cushion, pH 6) then placed inside the microwave for 20 min for heat induction of antigen recovery. A blocking solution of phosphate-buffered saline (PBS) and 10% normal goat serum is added to block nonspecific protein binding. Using PBS, the diluted primary antibody (1:200) was added to the slides, and they were incubated for 30 min, then few drops of streptavidin peroxidase were added to the slides, waiting for 20 min, then washed with PBS for 5 min. The chromogen Diaminobenzidine (DAB) (Dakopatts, Glostrup, Denmark) was added to the slides; then washed with distilled water. Lastly, the slides were counterstained with Harris hematoxylin (H), dehydrated and cover slipped. Negative controls were run consistently in parallel by skipping the primary antibody.

β-Arrestin2 immunohistochemical staining appeared as brownish discoloration of the cellular cytoplasm. The primary antibody was the monoclonal antibody to β-arrestin2 (β-arrestin 2 (C16D9) Rabbit mAb, Cat No. #3857, Cell Signaling, Danvers, USA).

β-Arrestin2 immuno-stained sections were morphometrically analyzed. The area percentage of immune reaction to β-arrestin2 was measured within ventricular sections of three rats in each group at a magnification ×100 using ImageJ v.1.51d (NIH & LOCI, Wisconsin University, USA). Briefly, stain-positive (brown-colored) areas were selected, masked by red binary coloring and measured on a standard measurement frame.

### Pooling of samples

Samples of each group were randomly pooled in three pools (Schisterman and Vexler 2008). Each pool consists of either one, two or three samples based on the number of animals that survived to the end of the experiment

### Statistical analysis

All data were presented as the mean ± standard error of the mean (SEM). Group means were compared by one-way ANOVA followed by Bonferroni post-test for selected pairs as showed using GraphPad Prism v. 5 (GraphPad Software, Inc., La Jolla, CA, USA). A *P* < 0.05 (two-tailed) was considered significant for all tests.

## Results

### Effect on mortality rate %, heart weight normalized to tibial length and blood markers of cardiac injury

**Fig. 2:**
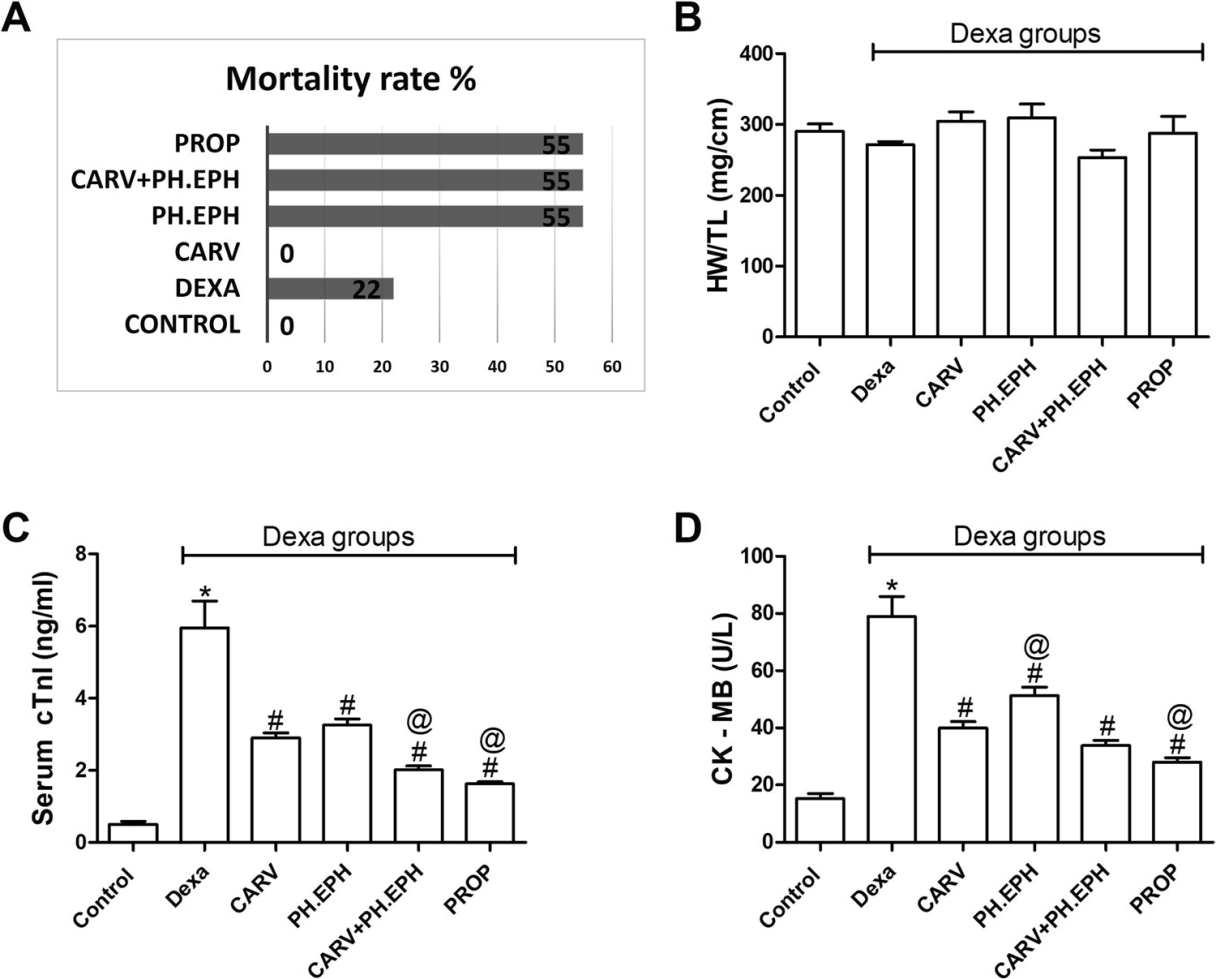
Changes in mortality rate %, heart weight normalized to tibial length and blood markers of cardiac injury. Graphical presentation of mortality rate % (**a**), heart weight normalized to tibial length (HW/TL) (**b**), serum level of cardiac troponin I (cTnI) (**c**) and creatine kinase–myoglobin (CK–MB) (**d**). Control: Rats received vehicle for 7 days. Dexa: Rats received dexamethasone (10 mg/kg, S.C.) for 7 days. CARV: Rats received carvedilol (10 mg/kg, I.P.) then dexamethasone 2 hours later for 7 days. PH.EPH: Rats received phenylephrine (1 mg/kg, I.P.) then dexamethasone 2.5 hours later for 7 days. CARV+PH.EPH: Rats received phenylephrine then carvedilol 30 minutes later then dexamethasone 2 hours later for 7 days. PROP: Rats received propranolol (30 mg/kg, I.P.) then dexamethasone 2 hours later for 7 days. Statistical analysis was performed using one-way ANOVA followed by Bonferroni post-test for selected pairs. Values are expressed as mean ± S.E.M. *n* = 9 for the control and CARV groups. *n* = 7 for the Dexa group. *n* = 4 for the PH.EPH, CARV+PH.EPH and PROP groups. Blood samples of each group were randomly pooled in three pools. * *P* < 0.05 vs control; # *P* < 0.05 vs Dexa; @ *P* < 0.05 vs CARV

Rats of the control and carvedilol groups showed 0% mortality, while those of the dexamethasone, phenylephrine, phenylephrine plus Carvedilol and propranolol groups showed 22%, 55%, 55% and 55% mortality, respectively (Fig. 2a). No significant changes were seen in the heart weights normalized to tibial lengths among all examined groups (Fig. 2b).

Subcutaneous injection of 10 mg/kg dexamethasone for 7 days in rats significantly increased serum levels of cTnI (Fig. 2c) and CK–MB (Fig. 2d) when compared to the control group. On the other hand, administration of either carvedilol or phenylephrine for 7 days concurrently with dexamethasone significantly decreased the serum levels of cTnI (Fig. 2c) and CK–MB (Fig. 2d) when compared to the dexamethasone group. The joint administration of phenylephrine and carvedilol concurrently with dexamethasone significantly decreased the serum levels of cTnI (Fig. 2c), while a nonsignificant decrease was detected with CK–MB (Fig. 2d) when compared to the carvedilol group. In addition, administration of propranolol concurrently with dexamethasone significantly decreased the serum levels of cTnI (Fig. 2c) and CK–MB (Fig. 2d) when compared to the carvedilol group.

### Effect on the cardiac levels of diacylglycerol (DAG), malondialdehyde (MDA) and Akt kinase activity

**Fig. 3:**
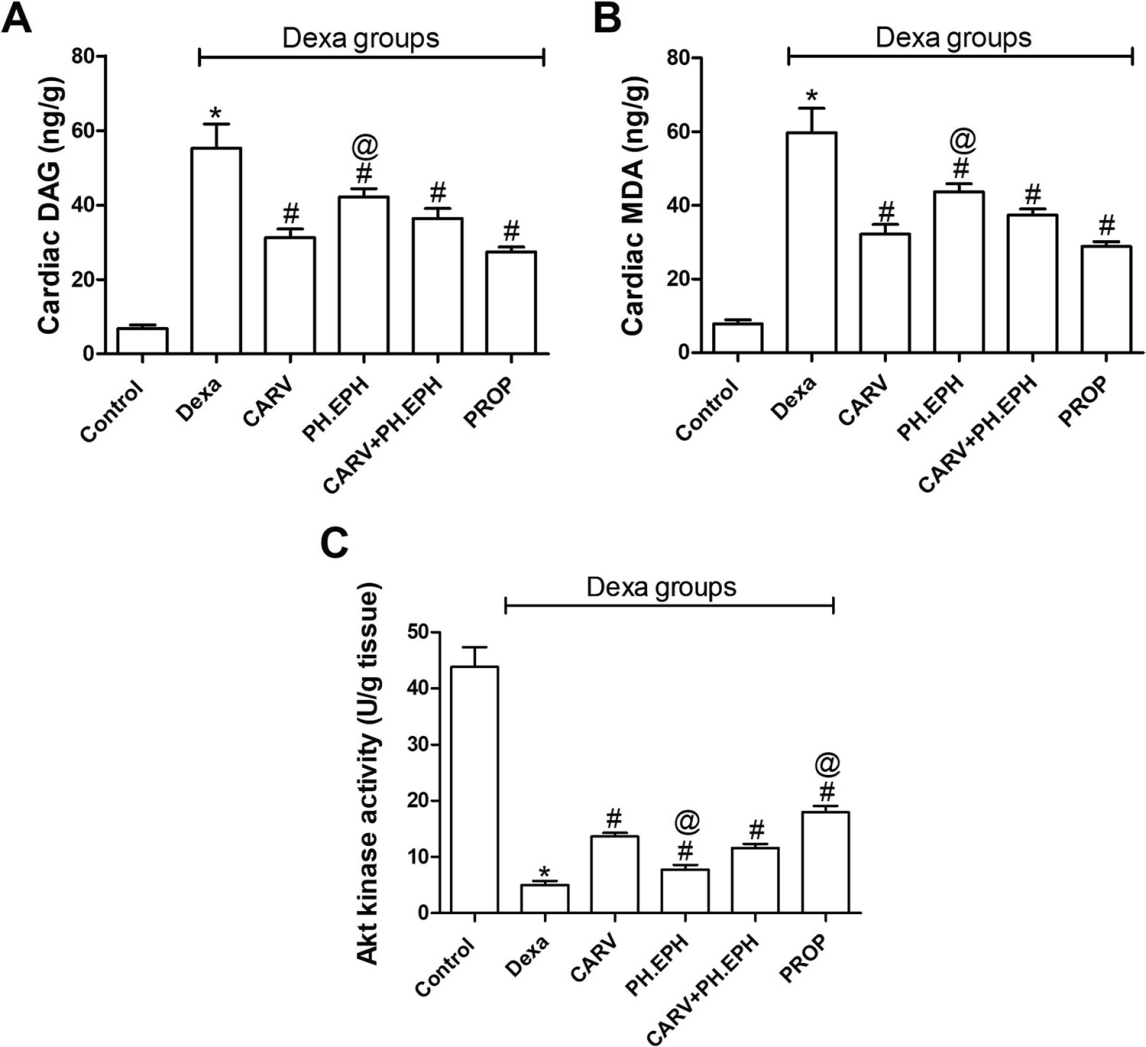
Changes in the cardiac levels of diacylglycerol (DAG), malondialdehyde (MDA) and Akt kinase activity. Graphical presentation of the DAG (**a**), MDA (**b**) and Akt kinase activity (**c**). Control: Rats received vehicle for 7 days. Dexa: Rats received dexamethasone (10 mg/kg, S.C.) for 7 days. CARV: Rats received carvedilol (10 mg/kg, I.P.) then dexamethasone 2 hours later for 7 days. PH.EPH: Rats received phenylephrine (1 mg/kg, I.P.) then dexamethasone 2.5 hours later for 7 days. CARV+PH.EPH: Rats received phenylephrine then carvedilol 30 minutes later then dexamethasone 2 hours later for 7 days. PROP: Rats received propranolol (30 mg/kg, I.P.) then dexamethasone 2 hours later for 7 days. Statistical analysis was performed using one-way ANOVA followed by Bonferroni post-test for selected pairs. Values are expressed as mean ± S.E.M. *n* = 9 for the control and CARV groups. *n* = 7 for the Dexa group. *n* = 4 for the PH.EPH, CARV+PH.EPH and PROP groups. Samples of each group were randomly pooled in three pools. * *P* < 0.05 vs control; # *P* < 0.05 vs Dexa; @ *P* < 0.05 vs CARV

As shown in Fig. 3, dexamethasone treatment significantly increased the cardiac levels of DAG (Fig. 3a) and MDA (Fig. 3b) when compared to the control group. Administration of either carvedilol, phenylephrine or propranolol concurrently with dexamethasone significantly decreased the cardiac levels of DAG (Fig. 3a) and MDA (Fig. 3b). However, the joint administration of carvedilol with phenylephrine did not significantly change the cardiac levels of both DAG and MDA when compared to the carvedilol group.

On the other hand, dexamethasone treatment significantly decreased the cardiac levels of Akt kinase activity (Fig. 3c) when compared to the control group. Administration of either carvedilol, phenylephrine or propranolol concurrently with dexamethasone significantly increased the cardiac Akt kinase activity (Fig. 3c) when compared to the dexamethasone group. In the same context, the joint administration of carvedilol and phenylephrine concurrently with dexamethasone did not change the cardiac Akt kinase activity when compared to the carvedilol group.

### Carvedilol, phenylephrine and propranolol reduced the dexamethasone-induced upregulation of cardiac profibrotic signals

**Fig. 4:**
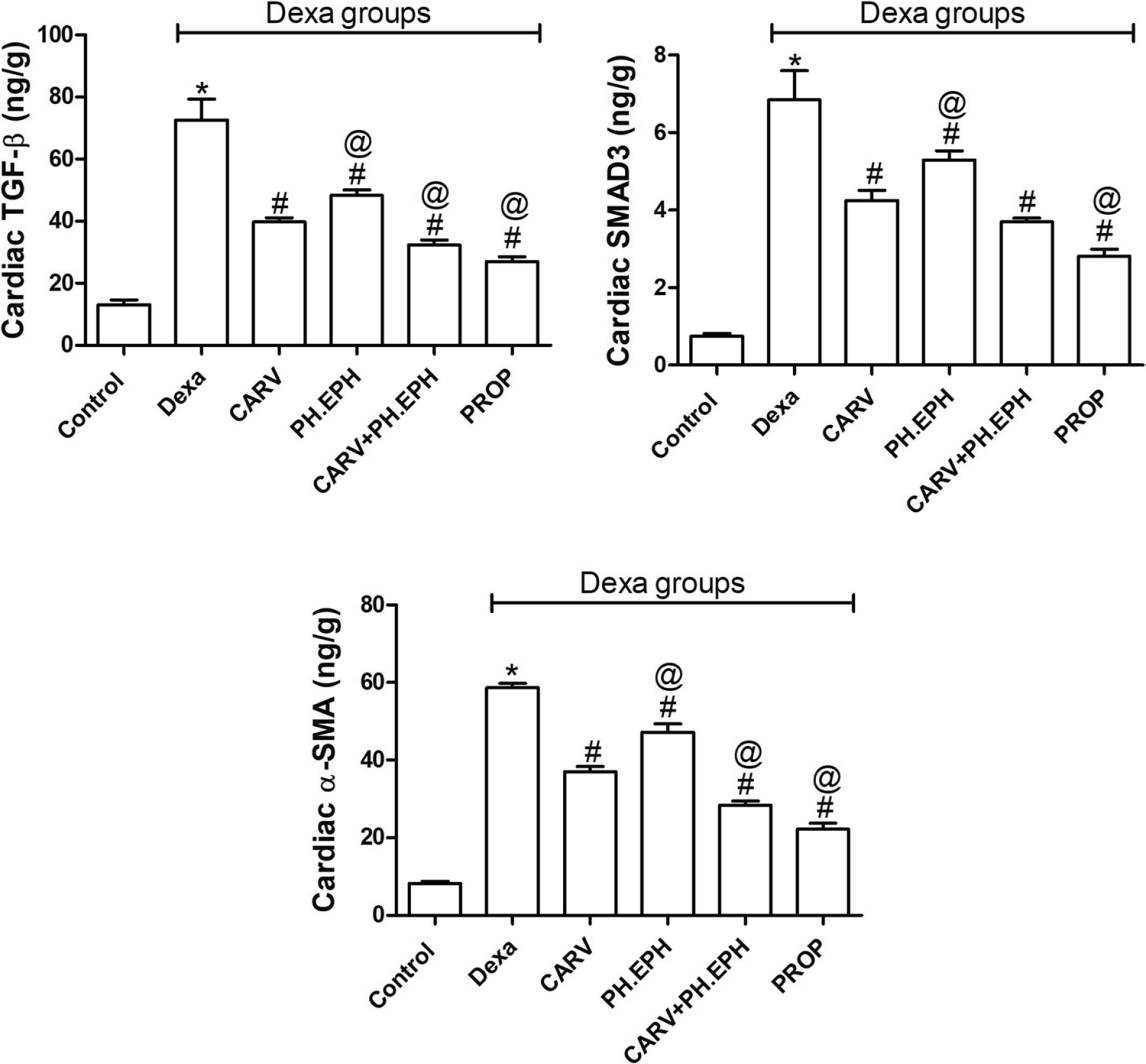
Changes in the cardiac levels of profibrotic signals. Graphical presentation of transforming growth factor-β (**a**), SMAD3 (**b**) and alpha smooth muscle actin (α-SMA) **(C)**. Control: Rats received vehicle for 7 days. Dexa: Rats received dexamethasone (10 mg/kg, S.C.) for 7 days. CARV: Rats received carvedilol (10 mg/kg, I.P.) then dexamethasone 2 hours later for 7 days. PH.EPH: Rats received phenylephrine (1 mg/kg, I.P.) then dexamethasone 2.5 hours later for 7 days. CARV+PH.EPH: Rats received phenylephrine then carvedilol 30 minutes later then dexamethasone 2 hours later for 7 days. PROP: Rats received propranolol (30 mg/kg, I.P.) then dexamethasone 2 hours later for 7 days. Statistical analysis was performed using one-way ANOVA followed by Bonferroni post-test for selected pairs. Values are expressed as mean ± S.E.M. *n* = 9 for the control and CARV groups. *n* = 7 for the Dexa group. *n* = 4 for the PH.EPH, CARV+PH.EPH and PROP groups. Samples of each group were randomly pooled in three pools. * *P* < 0.05 vs control; # *P* < 0.05 vs Dexa; @ *P* < 0.05 vs CARV

As shown in Fig. 4, dexamethasone treatment significantly increased the cardiac levels of TGF-β (Fig. 4a), Smad3 (Fig. 4b) and α-SMA (Fig. 4c) when compared to the control group. Administration of either carvedilol, phenylephrine or propranolol concurrently with dexamethasone significantly decreased the cardiac levels of TGF-β (Fig. 4a), Smad3 (Fig. 4b) and α-SMA (Fig. 4c) when compared to the dexamethasone group. On the other hand, the joint administration of carvedilol and phenylephrine concurrently with dexamethasone did not significantly change the cardiac Smad3 level but significantly decreased the cardiac TGF-β (Fig. 4a) and α-SMA level (Fig. 4c) when compared to the carvedilol group.

### Changes in the histopathology of the cardiac tissue using the hematoxylin and eosin stain

**Fig. 5:**
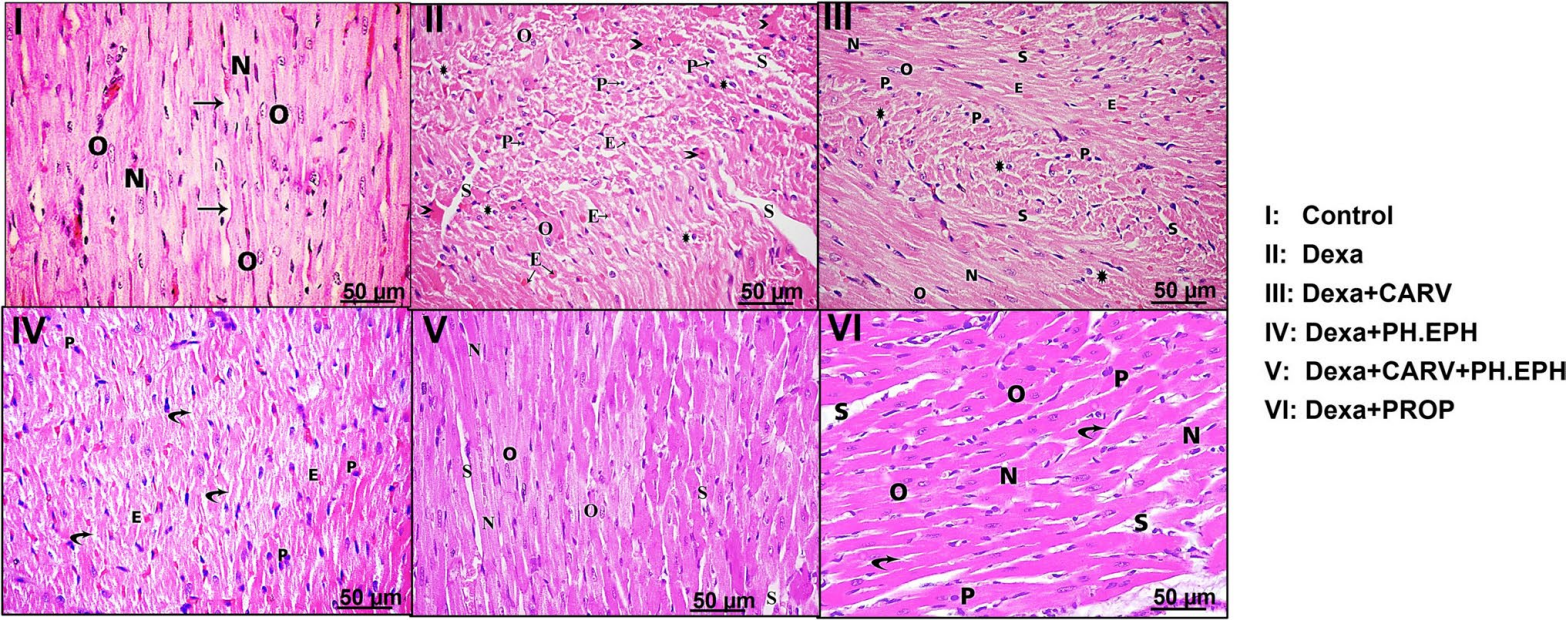
Histopathological changes in the cardiac ventricles stained with hematoxylin and eosin (H&E) stain (×400). I-VI: Representative photomicrographs of ventricular tissues. O: Central oval vesicular nuclei of the cardiac myocytes; Arrows: Small blood capillaries; N: elongated nuclei of interstitial cells; S: Abnormal interstitial separations; Arrowheads: Hyper-eosinophilia; *: Cytoplasmic vacuolation; P: Peripheral small dark nuclei; E: Extravasated blood cells; Curved arrows: Distortion of cardiac muscle striations. Control: Rats received vehicle for 7 days. Dexa: Rats received dexamethasone (10 mg/kg, S.C.) for 7 days. CARV: Rats received carvedilol (10 mg/kg, I.P.) then dexamethasone 2 hours later for 7 days. PH.EPH: Rats received phenylephrine (1 mg/kg, I.P.) then dexamethasone 2.5 hours later for 7 days. CARV+PH.EPH: Rats received phenylephrine then carvedilol 30 minutes later then dexamethasone 2 hours later for 7 days. PROP: Rats received propranolol (30 mg/kg, I.P.) then dexamethasone 2 hours later for 7 days

As shown in Fig. 5, a photomicrograph of the ventricular tissue of control rat (Fig. 5I) shows regular arrangement of longitudinal striated branching cardiac muscle fibers with acidophilic sarcoplasm and central oval vesicular nuclei (O) of the cardiac myocytes. In addition, small blood capillaries (*arrows*) and elongated nuclei of interstitial cells (N) are seen. Dexamethasone treatment-induced histopathological changes in the cardiac muscles such as distortion of cardiac muscle striations with some abnormal interstitial separations (S) (Fig. 5II). Furthermore, cardiac tissue of dexamethasone-treated rats showed hyper-eosinophilia (arrowheads), cytoplasmic vacuolations (astrix), peripheral small dark nuclei (pyknotic nuclei) (P), areas of myocytes with central oval vesicular nuclei (O) and extravasated blood cells (E). These histopathological changes reflect degeneration and cardiomyocyte apoptosis and impaired blood vessels integrity.

Co-administration of carvedilol and phenylephrine as well as their separate treatment markedly improved the cardiac tissue integrity. This was shown by marked reduction in the interstitial separations, distortion of cardiac muscle striations, hyper-eosinophilia and pyknotic nuclei (Fig. 5III, IV and V).

In the same context, administration of propranolol markedly reduced the dexamethasone-induced cardiac histopathological changes. This was shown by marked reduction in the interstitial separations, distortion of cardiac muscle striations, cytoplasmic vacuolations, pyknotic nuclei and hyper-eosinophilia (Fig. 5VI).

### No significant changes were seen in the cardiac fibrosis area percentage in all examined groups

**Fig. 6:**
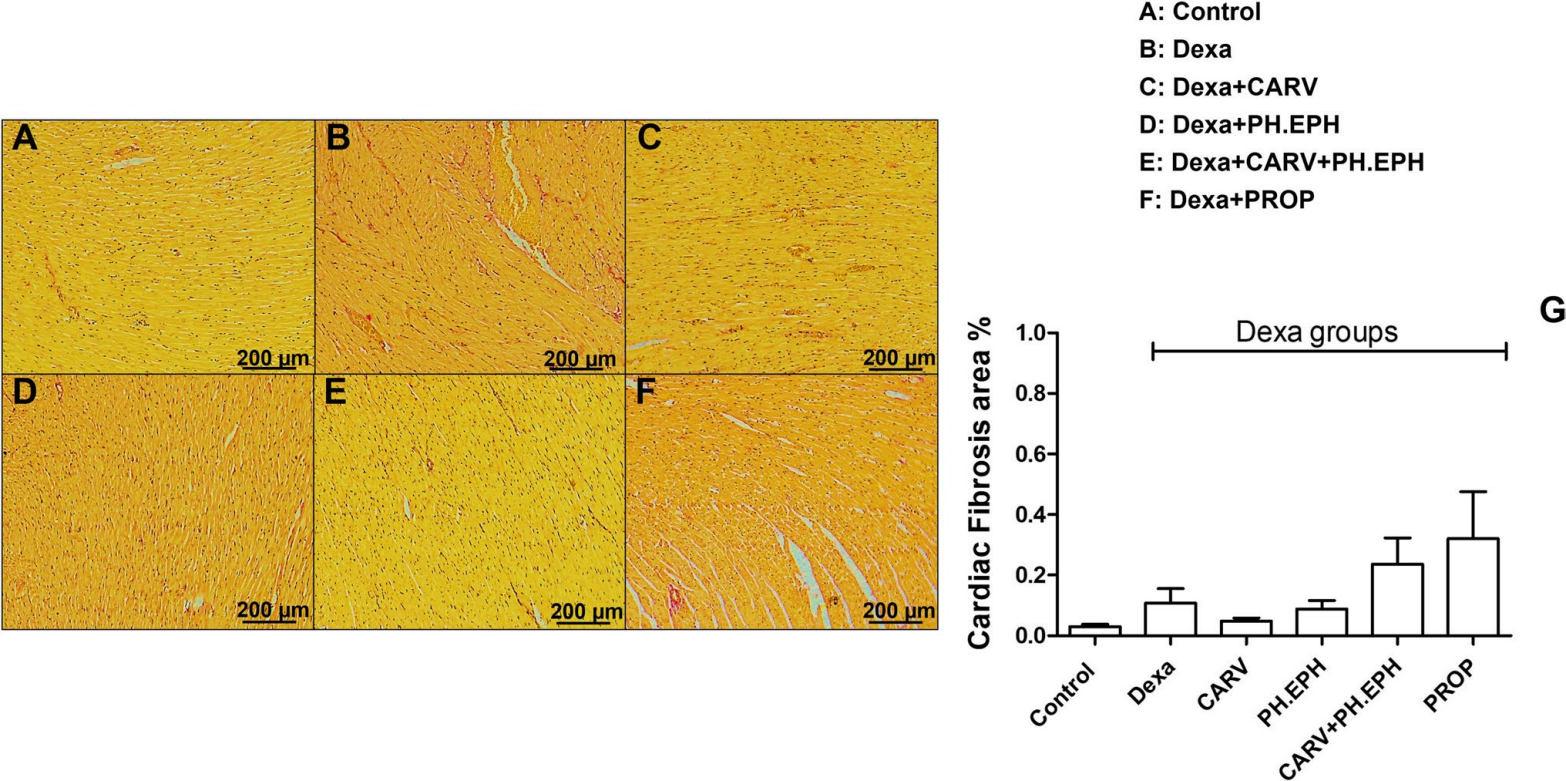
Changes in collagen deposits in the cardiac ventricles stained with Sirius red (×100). A–F: Representative photomicrographs of ventricular tissues. G: Graphical presentation of the fibrosis area percentage. Control: Rats received vehicle for 7 days. Dexa: Rats received dexamethasone (10 mg/kg, S.C.) for 7 days. CARV: Rats received carvedilol (10 mg/kg, I.P.) then dexamethasone 2 hours later for 7 days. PH.EPH: Rats received phenylephrine (1 mg/kg, I.P.) then dexamethasone 2.5 hours later for 7 days. CARV+PH.EPH: Rats received phenylephrine then carvedilol 30 minutes later then dexamethasone 2 hours later for 7 days. PROP: Rats received propranolol (30 mg/kg, I.P.) then dexamethasone 2 hours later for 7 days. Statistical analysis was performed using one-way ANOVA followed by Bonferroni post-test for selected pairs. Values are expressed as mean ± S.E.M. *n* = 3

As shown in Fig. 6, no significant changes were seen in the interstitial cardiac fibrosis area percentage in all examined groups. Interstitial cardiac collagen deposits were minimal in all groups (<0.5%).

### Phenylephrine and propranolol significantly decreased the cardiac β-arrestin2 level in dexamethasone-treated rats

**Fig. 7:**
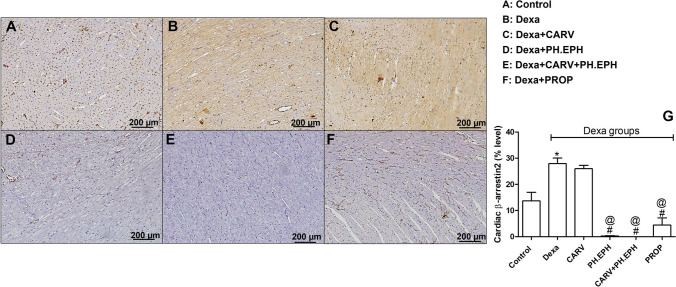
Changes in cardiac β-arrestin2 level. A–F: Representative photomicrographs of immuno-stained ventricular tissues (×100). G: Graphical presentation of the β-arrestin2 level percentage. Control: Rats received vehicle for 7 days. Dexa: Rats received dexamethasone (10 mg/kg, S.C.) for 7 days. CARV: Rats received carvedilol (10 mg/kg, I.P.) then dexamethasone 2 hours later for 7 days. PH.EPH: Rats received phenylephrine (1 mg/kg, I.P.) then dexamethasone 2.5 hours later for 7 days. CARV+PH.EPH: Rats received phenylephrine then carvedilol 30 minutes later then dexamethasone 2 hours later for 7 days. PROP: Rats received propranolol (30 mg/kg, I.P.) then dexamethasone 2 hours later for 7 days. Statistical analysis was performed using one-way ANOVA followed by Bonferroni post-test for selected pairs. Values are expressed as mean ± S.E.M. *n* = 3. * *P* < 0.05 vs control; # *P* < 0.05 vs Dexa; @ *P* < 0.05 vs CARV

As shown in Fig. 7, dexamethasone treatment significantly increased the cardiac levels of β-arrestin2 when compared to the control group (Fig. 7a, b and g). On the other hand, carvedilol treatment did not significantly decrease the cardiac β-arrestin2 level when compared to the dexamethasone group (Fig. 7c and g). Administration of either phenylephrine or propranolol concurrently with dexamethasone significantly decreased the cardiac levels of β-arrestin2 ((Fig. 7d), and (Fig. 7f), respectively, and Fig. 7g) when compared to the dexamethasone group. Furthermore, the joint administration of carvedilol and phenylephrine concurrently with dexamethasone significantly decreased the cardiac β-arrestin2 level (Fig. 7e and g) when compared to the carvedilol group.

## Discussion

In the current study, we investigated the cardiotoxic effects of dexamethasone-high-dose, the cardioprotective effects of carvedilol and the role of α1-adrenergic receptor in mediating these effects. Dexamethasone (10 mg/kg) was injected S.C. for 7 days. This model was previously used as a model of insulin resistance and metabolic syndrome (Mahendran and Devi 2001). Results of the current work showed that, dexamethasone treatment significantly increased serum levels of both cTnI and CK–MB when compared to the control group. cTnI is a specific type of troponin proteins that is preferentially expressed in cardiomyocytes and is released into the circulation after cardiomyocytes damage (Soetkamp et al. 2017). Like cTnI, CK–MP is highly expressed in cardiac muscles and is released into the circulation after cardiac injury (Abdel-Wahab et al. 2014).

In harmony with the findings of the current study, screening of a large international healthcare data network performed in 2013 classified corticosteroids as one of the primary causes of drug-induced acute myocardial infarction (Coloma et al. 2013). The cardiotoxic effects of dexamethasone were highly correlated with their atherogenic effects, sensitization of adrenergic receptors and induction of dyslipidemia (Ferrau and Korbonits 2015). In the same context, an earlier report showed that using dexamethasone (30 mg daily) for 4 days caused acute myocardial infarction in a 75-year-old woman with a medical history of diabetes and dyslipidemia (Shokr et al. 2016). Notably, the dexamethasone-dose used in our experiment is equivalent to 113 mg in humans weighting 70 kg.

Furthermore, dexamethasone treatment significantly increased cardiac levels of DAG and MDA when compared to the control group. DAG is a lipid mediator that promotes upregulation of the apoptotic and profibrotic pathways in addition to the production of reactive oxygen species (ROS) (Volpe et al. 2018). On the other hand, MDA is a lipid peroxidation product which is widely used as a marker of oxidative stress (Del Rio et al. 2005). Notably, it has been previously reported that dexamethasone can cause cardiac dysfunction by the activation of angiotensin II pathway and the induction of myocardial lipid peroxidation (Qi and Rodrigues 2007).

Consistent with earlier changes, dexamethasone treatment significantly decreased the kinase activity of cardiac Akt when compared to the control group. Akt, also known as protein kinase B (PKB), regulates distinct intracellular pathways such as glucose uptake and apoptosis (Mohamed et al. 2016). Decreased kinase activity of cardiac Akt is consistent with the dexamethasone-induced cardiomyocyte apoptosis. It has been previously reported that dexamethasone can directly downregulate the Akt activity in C2C12 cells (Kim et al. 2016). In addition, dexamethasone (2 mg/kg, IP.) treatment for 7 days has been found to reduce Akt level in the vascular smooth muscles (Araujo et al. 2020).

The current study also showed that dexamethasone treatment significantly increased the cardiac levels of TGFβ, Smad3 and α-SMA proteins when compared to the control group. TGFβ is a highly potent profibrogenic cytokine (Gharee-Kermani and Pham 2001). Increased TGFβ level is consistent with increased myocardial apoptosis and oxidative stress. Oxidative stress can activate latent TGFβ and increase its expression. Noteworthy, TGFβ itself can increase ROS production (Liu and Desai 2015). Activated TGFβ upregulates Smad3 and mediates its nuclear translocation which in turn promotes α-SMA expression (Yamazaki et al. 2014; Taiyab et al. 2019). Moreover, Smad3 can mediate the apoptotic effects of TGFβ (Millet and Zhang 2007).

Following the biochemical changes, histopathological examination of the cardiac tissue revealed marked myocardial degeneration and cardiomyocyte apoptosis in the dexamethasone group compared to the control group. In addition, immunohistochemical examination of the cardiac tissue showed significant increase in the β-arrestin2 level in the dexamethasone group compared to the control group. This finding is consistent with earlier reports which showed upregulation of cardiac β-arrestin2 in experimental models of cardiac ischemia-reperfusion injury (Wang et al. 2017) and drug-induced cardiac fibrosis (Nakaya et al. 2012) implicating a role for β-arrestin2 in mediating myocardial degeneration and cardiomyocyte apoptosis. On the contrary, dexamethasone group showed minimal and nonsignificant increase in the cardiac interstitial fibrosis area % when compared to the control group. An interpretation of this finding is that duration of experiment is short, and a longer time may be needed to record significant and marked increases in collagen deposits.

Carvedilol is a third-generation β-blocker with α1-blocking effects (Frishman 1998). In addition, carvedilol has antioxidant and anti-inflammatory effects and can uniquely activate β-arrestin signaling (Wisler et al. 2007). Because oxidative stress and sensitization of adrenergic receptors play a significant role in the cardiotoxic actions of dexamethasone, we expected the presence of a potential cardioprotective effect of carvedilol against dexamethasone-induced cardiotoxicity.

Consistent with our expectations, carvedilol significantly reduced serum levels of cTnI and CK–MB when compared to the dexamethasone group. These findings confirm protection against dexamethasone-induced cardiac injury. The cardioprotective actions of carvedilol were associated with significant reduction in the cardiac levels of DAG and MDA when compared to the dexamethasone group, reflecting mitigation of oxidative stress. Moreover, carvedilol significantly increased the kinase activity of cardiac Akt and decreased the cardiac levels of TGF-β, Smad3 and α-SMA when compared to the dexamethasone group, showing amelioration of cardiac apoptosis and fibrosis. In harmony with the biochemical changes, histopathological examination of the cardiac tissue using H&E stain showed marked reduction in the myocardial degeneration and cardiomyocyte apoptosis. On the contrary, carvedilol treatment showed slight and nonsignificant decrease in the β-arrestin2 level when compared to the dexamethasone group during immunohistochemical examination of the cardiac tissue. This may be attributed to the mixed carvedilol direct and indirect effects on β-arrestin2 in this group. It has been previously reported that carvedilol has cardioprotective effects in diabetic cardiomyopathy and cardiorenal syndrome type 4 by upregulating the cardiac levels of β-arrestin2 (Ibrahim et al. 2020; Mohamed et al. 2021). Carvedilol has a unique activity that can direct β-arrestin2 toward cardioprotective pathways in contrast to dexamethasone which may do the reverse. Therefore, despite that carvedilol slightly decreased the cardiac β-arrestin2 level in dexamethasone-treated rats, it is possible that carvedilol redirected the activity of β-arrestin2 toward the cardioprotective pathways.

The antioxidant, anti-inflammatory and the β-blocking effects of carvedilol in mediating cardiac protection were extensively investigated in earlier studies (Aronow 2010). In the present work, we investigated the role of α1-adrenergic receptor in mediating the cardioprotective effects of carvedilol. α1-Adrenergic receptor is expressed in both cardiomyocytes and vascular smooth muscles (Arthur et al. 2001). Activation of cardiac α1-adrenergic receptor inhibits apoptosis independent of blood pressure (Woodcock et al. 2008). On the other hand, activation of the vascular α1-adrenergic receptor causes vasoconstriction and hypertension, increasing the workload on the heart which promotes myocardial apoptosis (Piascik and Perez 2001).

To clarify the role of α1-adrenergic receptor, we used carvedilol in combination with phenylephrine. Phenylephrine is a competitive α1-adrenergic receptor agonist which can compete with carvedilol on the binding to this receptor (Jensen et al. 2011). Interestingly, phenylephrine alone significantly decreased the serum levels of cardiac injury markers and significantly reduced the cardiac oxidative stress, apoptosis, profibrotic signals and histopathological changes when compared to the dexamethasone group. However, the cardioprotective effects of phenylephrine were weaker than those of carvedilol. Following our findings, an earlier study showed that phenylephrine inhibits neonatal cardiomyocyte apoptosis secondary to hypoxia and serum deprivation by preventing the downregulation of Bcl-2 and Bcl-x, enhancing the activity of PI3K/Akt pathway and inhibiting the activity of caspase-9 (Zhu et al. 2000). Moreover, it has been reported that activation of cardiac α1-adrenergic receptor protects the heart independent of blood pressure (Woodcock et al. 2008). On the contrary, the blocking of α1-adrenergic receptor worsens heart failure even in hypertension (Group 2000; Piller et al. 2002).

Consistent with earlier findings, concomitant use of phenylephrine and carvedilol in dexamethasone-treated rats potentiated the reduction in the serum markers of cardiac injury (cTnI and CK–MB) when compared to either carvedilol or phenylephrine single treatment. Similar changes were seen in the cardiac levels of TGF-β, Smad3, α-SMA and β-arrestin2 as well as in the cardiac histopathology. On the contrary, the changes in the cardiac levels of DAG, MDA and kinase activity of Akt revealed antagonism between carvedilol and phenylephrine. All together, these results support more the cardioprotective effects secondary to the activation of the α1-adrenergic receptor.

Confirming earlier findings, propranolol treatment significantly decreased the serum markers of cardiac injury when compared to both the dexamethasone and the carvedilol groups. Notably, the propranolol dose used in the current study is pharmacologically equivalent to that of carvedilol based on their anti-hypertensive actions (James et al. 1992). Therefore, the recorded changes are independent of blood pressure variations. Propranolol is a non-selective β-blocker without activity on the α1-adrenergic receptor. So that, cardiac α1-adrenergic receptors stay activated by endogenous catecholamines. Consistent with the changes in the serum markers, propranolol significantly decreased the cardiac levels of TGF-β, Smad3, α-SMA and β-arrestin2 as well as it improved the histopathological changes when compared to both the dexamethasone and the carvedilol groups. On the contrary, the changes in the DAG, MDA and kinase activity of Akt were nonsignificant when compared to the carvedilol group. These findings may point to a superior role for the TGF-β/Smad3/α-SMA pathway in this model.

Despite the higher cardioprotective effects of propranolol when compared to the carvedilol group, the mortality rate was higher in this group (55%) than that of carvedilol (0%). It is possible that, cause of death in this group is not related to the myocardial injury. This point needs further investigations.

Also, although the doses of carvedilol and propranolol used in the current study are pharmacologically equivalent regarding their effect on the blood pressure, recording changes in blood pressure in the examined groups would strengthen and support the obtained results. Therefore, this point is considered a study limitation.

In conclusion, dexamethasone-high-dose upregulates cardiac oxidative stress, apoptotic and profibrotic signals and induces myocardial injury. Carvedilol, phenylephrine and propranolol improve dexamethasone-induced myocardial injury. Blocking the α1-adrenergic receptor by carvedilol attenuates its cardioprotective effects.

## Data Availability

All the data of this study are transparent and available upon request.
